# Increased CSF Soluble TREM2 Concentration in Patients With Neurosyphilis

**DOI:** 10.3389/fneur.2020.00062

**Published:** 2020-02-05

**Authors:** Wurong Li, Haoxiao Chang, Wenqing Wu, Dongmei Xu, Meijuan Jiang, Junhua Gao, Yuming Huang, Yun Xu, Linlin Yin, Xinghu Zhang

**Affiliations:** ^1^Department of Neurology, Beijing Ditan Hospital, Capital Medical University, Beijing, China; ^2^Department of Neurology, Beijing Tiantan Hospital, Capital Medical University, Beijing, China; ^3^China National Clinical Research Center for Neurological Diseases, Beijing, China

**Keywords:** neurosyphilis, microglia, cerebrospinal fluid, soluble triggering receptor expressed on myeloid cells 2, neurofilament light proteins

## Abstract

**Objective:** To explore cerebrospinal fluid (CSF) levels of soluble triggering receptor expressed on myeloid cells 2 (sTREM2) and neurofilament light proteins (NFL) in patients with neurosyphilis (NS).

**Methods:** We enrolled 71 NS patients (41 early-NS and 30 late-NS patients) and 20 syphilis but non-NS patients whose CSF samples were collected. The CSF levels of the microglial activation biomarker sTREM2 and neuronal injury biomarker NFL were measured using ELISA.

**Results:** CSF sTREM2 levels were significantly higher in NS patients compared to those in syphilis/non-NS patients (*p* < 0.001). In a subgroup analysis, the CSF sTREM2 levels elevated significantly in late-NS patients than those in early-NS patients (*p* < 0.001). The CSF sTREM2 levels in early-NS group were also significantly higher than those in syphilis/non-NS group (*p* = 0.024). Like CSF sTREM2, similar differences between groups were also found in CSF NFL. There was a moderate correlation between CSF sTREM2 and CSF NFL (*r* = 0.406, *p* < 0.001) in NS group.

**Conclusions:** CSF sTREM2 levels elevated in NS and peaked at the late stage, suggesting that CSF sTREM2 may be a useful marker to quantify microglia activation in NS and may play a role in the progression of NS. The positive correlation between CSF sTREM2 and CSF NFL indicates a linkage between microglial activation and neuronal injury in NS.

## Introduction

NS is a chronic infectious disease caused by *Treponema pallidum* invading the central nervous system (CNS), which mainly damages the meninges, blood vessels, and brain, and spinal cord parenchyma (1, 2). A large number of activated microglia were found in the brain autopsies of NS, demonstrating that the microglial activation was an important pathological feature of NS (3, 4), which was rarely studied in CSF samples of patients with NS.

The triggering receptor expressed on myeloid cells-2 (TREM2) is a cell surface receptor protein, which is mainly expressed in myelocytes (5). Most relevant for the brain is the microglial expression of TREM2, which promotes phagocytosis, suppresses Toll-like receptor-induced inflammatory cytokine production, and enhances anti-inflammatory cytokine transcription *in vitro* (6–8). Increased soluble TREM2 (sTREM2) levels in CSF have been found in HIV infection (8), multiple sclerosis (MS) (9, 10), and Alzheimer's disease (AD) (11, 12). However, over activation of microglia may result in neuronal loss and neuropil damage (3, 4). Neurofilament light proteins (NFL) are the most widely distributed and important component of neurofilament proteins (NFs) (13). The levels of CSF NFL can reflect the degree of neuronal injury of the CNS and severity of the disease to a certain extent. A correlation between microglial activation and neuronal injury was found in HIV infection (8). NS patients, especially in the late stage, have mental abnormalities, cognitive changes, and brain atrophy (1, 2), indicating that NS patients have a degree of neuronal loss and injury. We speculate that the microglial dysfunction is probably involved in the pathogenesis of mental and neurocognitive disorders in NS.

The aim of this study was to determine the levels of CSF sTREM2 in different stages of NS patients and to explore the relationship between CSF sTREM2 and CSF NFL, so as to better understand the neuropathogenesis and progression of NS.

## Materials and Methods

### Patients

Between May 2018 and June 2019, 71 NS patients and 20 syphilis but non-NS controls hospitalized in the neurology department of Beijing Ditan Hospital and Beijing Tiantan Hospital were analyzed in this study. We recorded the medical history, neurological symptoms and signs, and serum and CSF laboratory testing results. According to the guidelines of NS in the USA, Europe and related literatures (14–18), the criteria for the diagnosis of NS in our study included positive syphilis serologies and one or more of the followings: (a) positive CSF rapid plasma regain (RPR); (b) positive CSF *Treponema pallidum* particle agglutination (TPPA) and fluorescent treponemal antibody absorption (FTA-ABS), with increased CSF protein (>45 mg/dl) or white blood cells (WBC) (> 5/μl) in absence of other known causes of these abnormalities. All of the enrolled patients were HIV negative. The exclusion criteria were as follows: treatment with antibiotics within the last 1-month, other infectious diseases (e.g., HIV), neurodegenerative disease (e.g., AD) and autoimmune diseases (e.g., MS). The patients enrolled in the control group were a serofast status without neurological symptoms and signs and underwent lumber puncture to rule out neurosyphilis.

This study was approved by the Ethics Committee of Beijing Ditan Hospital Affiliated to Capital Medical University, Beijing, People's Republic of China and written informed consent was obtained from all participants.

The enrolled NS patients were divided into early-NS that occurred during the primary stage or secondary stage, including asymptomatic, meningeal and meningovascular NS and late-NS that occurred years to decades after the primary infection, including general paresis and tabes dorsalis (1, 4).

### Biomarker Measurement

CSF samples were immediately centrifuged and the supernatants were collected and stored at −80°C until the time of the biomarker assays. ELISA, to quantify CSF sTREM2 (ab224881, abcam) and NFL (CSB-E16094h, CUSA-BIO, Wuhan, China), was performed blinded to clinical information. All testing was performed according to the manufacturer's protocols and by the same technique.

### Statistical Analysis

Data were analyzed by SPSS software (IBM SPSS 25.0 version) or Prism (GraphPad software 7.0 version). CSF levels of sTREM2 and NFL were log_10_ transformed where appropriate to reduce skewness. Continuous data were compared with the non-parametric Mann-Whitney *U*-test when the data were non-parametric or independent two-sample *t*-test when the data were parametric. Gender differences were assessed by χ^2^-test. Pearson' test (for parametric data) and Spearman's test (for non-parametric data) were used to ascertain the associations for analysis of correlations. The significance level was established at a two-sided *p* < 0.05.

## Results

### Patients Characteristics

The clinical characteristics and concentrations of different CSF biomarkers were summarized in Table 1. A total of 71 NS patients (52 male and 19 female) and 20 syphilis but non-NS (9 male and 11 female) were studied. Forty-one patients with early-NS were enrolled, including asymptomatic NS (*n* = 34), meningeal NS (*n* = 1) and meningovascular NS (*n* = 6). In addition, 30 patients with late-NS were enrolled, including general paresis (*n* = 26) and tabes dorsalis (*n* = 4). The vast majority of patients received anti-syphilis treatment, and only 2 patients with paralytic dementia had no treatment history. The 30 cases of late-NS showed a variety of neurological symptoms and signs, including mental and behavior disorders (24/30), cognitive changes (23/30), sensory impairment (deduced vibration sense, Romberg sign, ataxia, painful polyradiculopathy, lighting pains) (8/30), parkinsonism (4/30), headache (3/30) and seizures (3/30). The symptoms of 41 cases of early-NS were asymptomatic (34/41), headache (1/41), stroke (6/41) and seizures (1/41).

**Table 1:** Characteristics of patients with the NS patients and Syphilis/non-NS controls.

**Subject details**	**NS (*****n*** **= 71)**		**Syphilis/non-NS** **(*n* = 20)**
	**Late-NS** **(*n* = 30)**	**Early-NS** **(*n* = 41)**	
Age, mean ± SD	50.73 ± 11.99	47.88 ± 12.48	44.31 ± 14.02
Sex ratio, n (M/F)	23/7	29/12	9 /11
Serum RPR titer, median (IQR)	1:16 (1:8–1:32)	1:8 (1:4–1:16)	1:4 (1:2–1:8)
CSF RPR titer, median (IQR)	1:1 (0–1:2)	0 (0–1:1)	0 (0–0)
CSF WBC count, median (IQR), /ul	8 (4-21)	7 (5-12)	3 (2-3)
CSF protein concentration, median (IQR), mg/dl	46.05 (34.6-69.43)	36.9 (26.6-53.8)	24.3 (18.0–32.6)
CSF sTREM2 (log_10_ pg/ml), median (IQR)	4.51 (4.34–4.65)	4.27 (4.11–4.41)	4.17 (4.02–4.25)
CSF NFL (log_10_ pg/ml), median (IQR)	1.73 (1.51–2.14)	1.48 (1.34–1.69)	1.22 (0.89–1.52)

*Data expressed as mean ± SD or median (IQR) as appropriate. NS, neurosyphilis; M, male; F, female; RPR, rapid plasma regain; CSF, cerebrospinal fluid; WBC, whiter blood cell; IQR, interquartile range; sTREM2, soluble Triggering receptor expressed on myeloid cells 2; NFL, neurofilament light protein; 0, negative; NA, not available*.

There was no significant difference in age and gender between the NS group and the syphilis/non-NS group (*p* = 0.65 and *p* = 0.29). The serum RRP titer was significantly higher in the NS group than in the syphilis/non-NS group (*p* < 0.001). As for CSF laboratory findings between the late-NS group and early-NS group, we found that the incidence of positive CSF RPR rate was significantly higher in the late-NS group than in the early-NS group (*p* < 0.001), but no significant difference of serum RPR titer, CSF protein concentration and CSF WBC was found between the two groups (*p* = 0.081, *p* = 0.115, and *p* = 0.670, respectively).

### CSF sTREM2 Levels Are Higher in NS Patients

**Figure 1:**
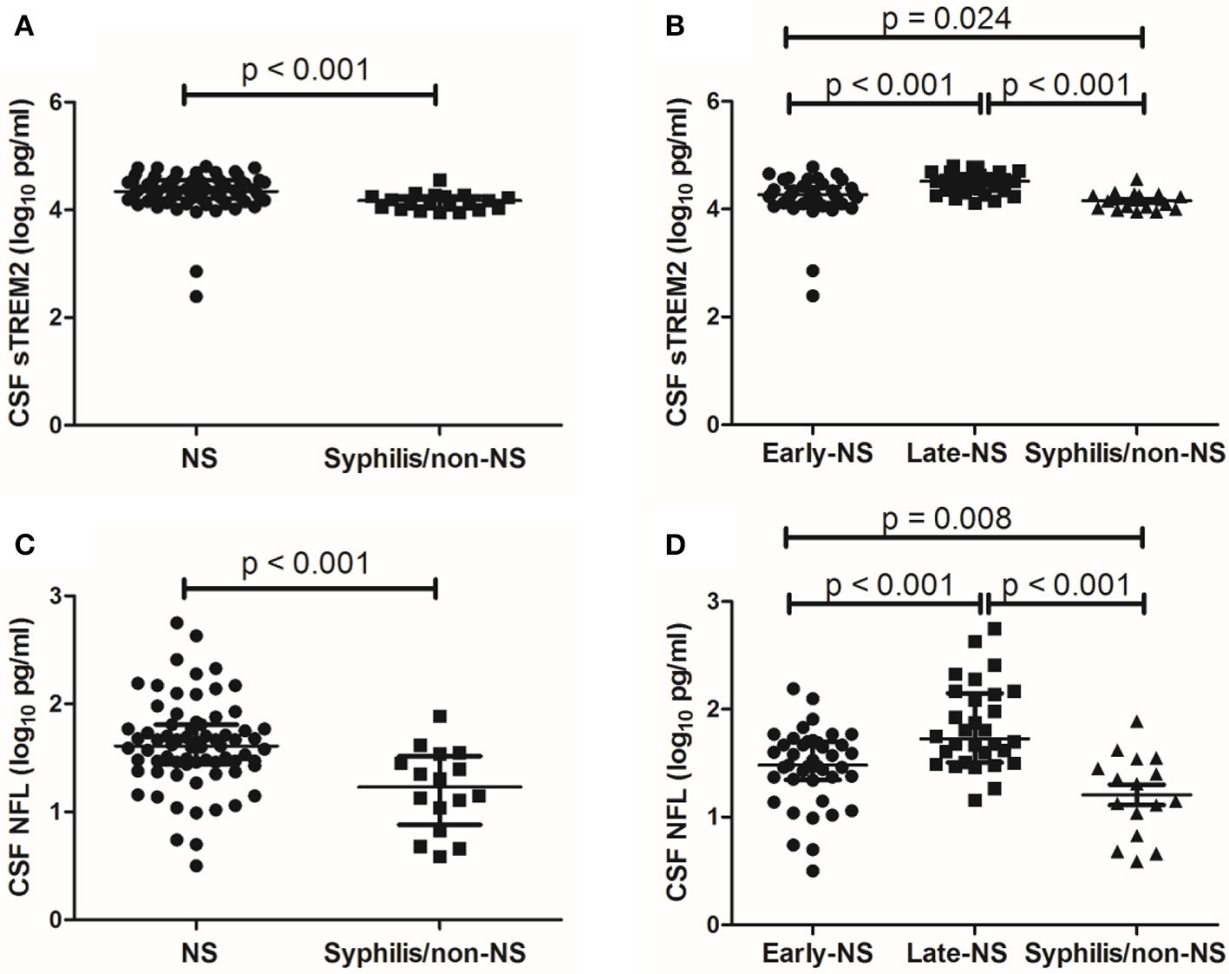
CSF levels of sTREM2 and NFL in NS and syphilis/non-NS patients. **(A)** Patients with NS had significantly higher levels of CSF sTREM2 (log_10_ pg/ml) than those with syphilis/non-NS. **(B)** The CSF sTREM2 (log_10_ pg/ml) levels in late-NS were significantly higher than in early-NS and syphilis/non-NS. The CSF sTREM2 (log_10_ pg/ml) levels in early-NS were also significantly higher than in syphilis/non-NS. **(C)** Patients with NS had significantly higher levels of CSF NFL (log_10_ pg/ml) than those with syphilis/non-NS. **(D)** The CSF NFL (log_10_ pg/ml) levels in late-NS were significantly higher than in early-NS and syphilis/non-NS. The CSF NFL (log_10_ pg/ml) levels in early-NS were also significantly higher than in syphilis/non-NS. NS, neurosyphilis; sTREM2, soluble triggering receptor expressed on myeloid cells 2; NFL, neurofilament light protein.

The median levels of CSF sTREM2 were 21793.98 (16273.05-35218.54) pg/ml in NS group and 14783.12 (10567.25-17894.95) pg/ml in non-NS group. CSF sTREM2 (log_10_) values in the NS group were significantly higher than those in the syphilis/non-NS group (*p* < 0.001; Figure 1A). In the NS subgroup analysis, the higher CSF sTREM2 levels were found in the late-NS group [32700.63 (21979.94-44562.75) pg/ml] than those in the early-NS group [18641.82 (12839.14-25737.66) pg/ml]. There was a statistically significant difference of CSF sTREM2 (log_10_) values between the two NS groups (*p* < 0.001; Figure 1B). The CSF sTREM2 (log_10_) values in the early-NS group were also significantly higher than those in the syphilis/non-NS group (*p* = 0.024; Figure 1B).

### CSF NFL Levels Are Higher in NS Patients

Like CSF sTREM2, we found similar differences between groups in CSF NFL. The statistical results for CSF NFL of group comparisons were shown in Figures 1C,D.

### Correlations of CSF sTREM2 Levels With CSF NFL and Age

**Figure 2:**
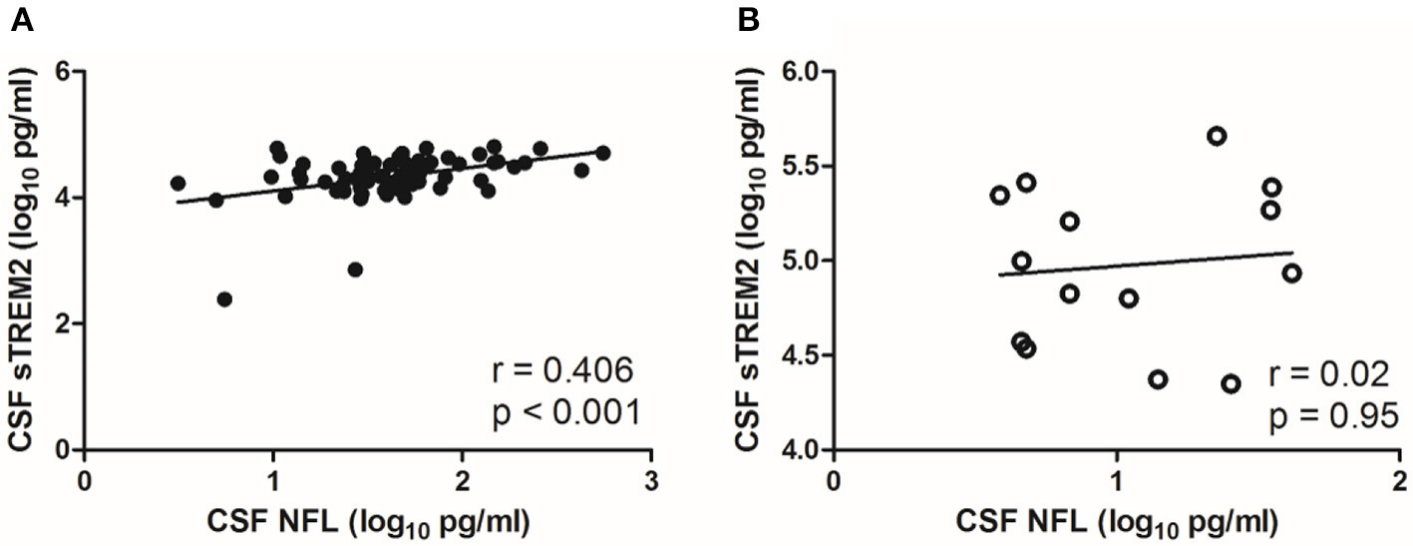
Correlations between CSF sTREM2 and CSF NFL in NS group and syphilis/non-NS group. **(A)** CSF sTREM2 (log_10_ pg/ml) levels were moderate correlated with CSF NFL (log_10_ pg/ml) in NS group. **(B)** No significant correlation was found between CSF sTREM2 (log_10_ pg/ml) and CSF NFL (log_10_ pg/ml) in syphilis/non-NS group. NS, neurosyphilis; CSF, cerebrospinal fluid; sTREM2, soluble triggering receptor expressed on myeloid cells 2; NFL, neurofilament light protein.

There was a moderate correlation between CSF sTREM2 (log_10_) values and CSF NFL (log_10_) (*r* = 0.406, *p* < 0.001; Figure 2A) in NS group, while no significant correlation was found between CSF sTREM2 (log_10_) and CSF NFL (log_10_) in the syphilis/non-NS group (*r* = 0.02, *p* = 0.95; Figure 2B).

**Figure 3:**
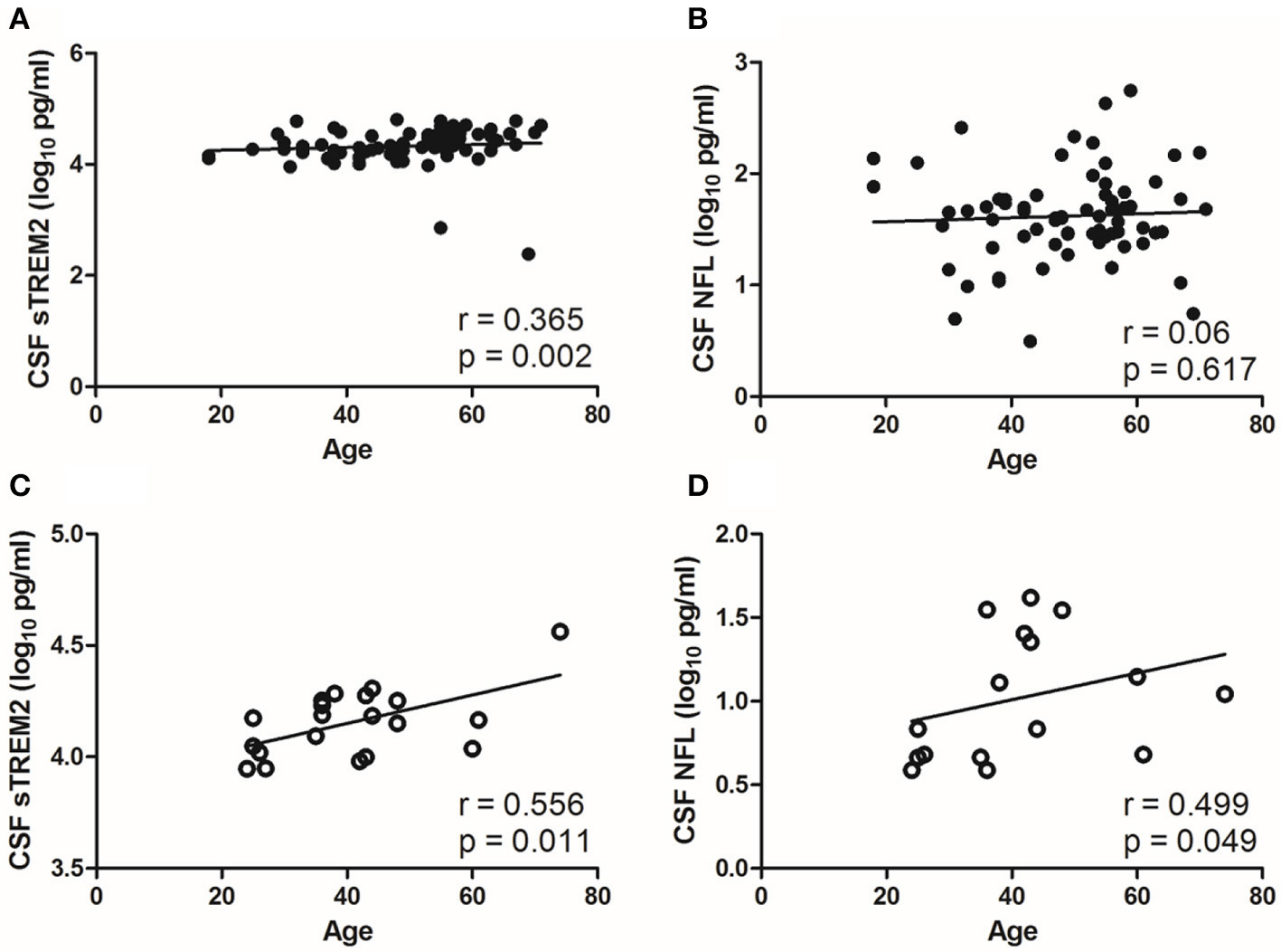
Correlations between CSF biomarkers and age in NS group and syphilis/non-NS group. **(A)** CSF sTREM2 (log_10_ pg/ml) levels were weak correlated with age in NS group. **(B)** No significant correlation was found between CSF NFL (log_10_ pg/ml) and age in NS group. **(C)** CSF sTREM2 (log_10_ pg/ml) levels were moderate correlated with age in syphilis/non-NS group. **(D)** CSF NFL (log_10_ pg/ml) levels were moderate correlated with age in syphilis/non-NS group. NS, neurosyphilis; CSF, cerebrospinal fluid; sTREM2, soluble triggering receptor expressed on myeloid cells 2; NFL, neurofilament light protein.

CSF sTREM2 (log_10_) levels were weak correlated with age in the NS group (*r* = 0.365, *p* = 0.002; Figure 3A), however, no significant correlation was found between CSF NFL (log_10_) and age in the NS group (*r*=0.06, *p* = 0.617; Figure 3B). The levels of CSF sTREM2 (log_10_) and CSF NFL (log_10_) were better correlated with age in syphilis/non-NS group (*r* = 0.556, *p* = 0.011 and *p* = 0.499, *p* = 0.049; Figures 3C,D).

### CSF sTREM2 Levels Are Higher in CSF RPR Positive Patients

**Figure 4:**
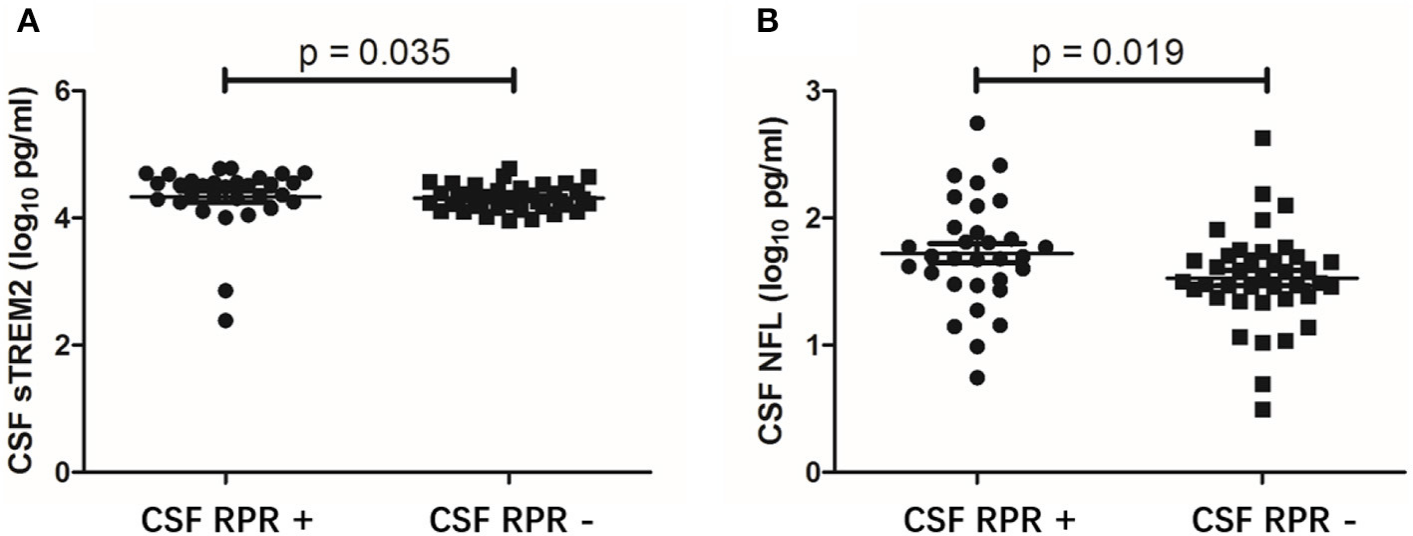
CSF levels of sTREM2 and NFL in NS patients with CSF RPR positive and CSF RPR negative. **(A)** The CSF sTREM2 (log_10_ pg/ml) levels in CSF RPR positive patients were significantly higher than in CSF RPR negative patients. **(B)** The CSF NFL (log_10_ pg/ml) levels in CSF RPR positive patients were significantly higher than in CSF RPR negative patients. CSF, cerebrospinal fluid; sTREM2, soluble triggering receptor expressed on myeloid cells 2; NFL, neurofilament light protein; RPR, rapid plasma regain.

To further evaluate the association between CSF sTREM2 and neuroinflammation, we divided the NS patients into CSF RPR positive group and CSF RPR negative group and compared the CSF sTREM2 levels between the two groups. Here, 43.7% (31/71) of our NS patients had CSF RPR positive, and the higher CSF sTREM2 levels were found in CSF RPR positive group. There were significant differences between CSF sTREM2 (log_10_) in the two groups (*p* = 0.035; Figure 4A). Like CSF sTREM2, CSF NFL levels in the CSF RPR positive group were significantly higher than those in the CSF RPR negative group (*p*=0.019; Figure 4B).

## Discussion

Microglia are important immunosurveillance cells in the CNS, and are important target cells for CNS infectious disease (19). Microglia activation may mediate neuroinflammation and cause dysfunction of neurons and neuropil in the disease (3, 4, 6, 7, 20, 21). TREM2 is a transmembrane protein selectively expressed by microglia in the brain, and its soluble variant (sTREM2) can be detected in CSF (9). In the present study, we investigated the changes of CSF sTREM2 levels at different stages of NS and explored the relationship between this microglial activation marker and neuronal injury marker NFL.

We found that the CSF sTREM2 levels elevated significantly in patients with early and late stages of NS compared to syphilis/non-NS patients, and peaked at late stage of NS. Researchers have found the pathophysiological function of TREM2 in animal models of different CNS diseases. In experimental autoimmune encephalitis, the expression levels of TREM2 on the surface of microglia increased significantly under inflammation. After blocking TREM2 expression, the inflammation and demyelination of the animals were aggravated (22). In AD mice, TREM2 silencing resulted in the increase expression of inflammatory factors, suggesting that TREM2 has an anti-inflammatory effect (20). Compared with non-inflammatory neurological diseases, the CSF sTREM2 levels increased in patients with MS and other inflammatory diseases, corroborating that sTREM2 reflects the neuroinflammatory process of the CNS (9). CSF RPR had an high diagnostic value in NS and was positively correlated with the inflammatory activity of the disease (15, 17, 18, 23). More patients in the late-NS group had positive CSF RPR, indicating persistent infection of *treponema pallidum* in the brain, which could cause and maintain neuroinflammation and tissue damage (24). The levels of CSF sTREM2 were higher in the NS group than those in the syphilis/non-NS group, as well as being higher in the CSF RPR positive group than those in the RPR negative group, suggesting that the expression of CSF sTREM2 was up-regulated under syphilis persistent infection to achieve an anti-inflammatory effect. One study of AD patients found that the expression of CSF sTREM2 increased, which was correlated with biomarkers of T-tau and P-tau, suggesting that the increase of inflammatory response was related to neurodegeneration (12). In our enrolled patients, 86.7% of late neurosyphilis were general paresis (26/30). The clinical manifestations of general paresis were very similar to those of AD. In addition, some studies found that general paresis was also associated with decreased CSF Aβ42 and elevated CSF t-tau and p-tau181 (25–28). CSF sTREM2 levels were higher in late-NS group, suggesting that sTREM2 may play an important role in the neurodegeneration and progression in the course of NS. Such a marker may be helpful to explore the pathogenesis of NS and provide a clinical usage for determining prognosis.

Another main finding of our study was that CSF NFL levels in the NS group were higher than those in the syphilis/non-NS group, reflecting ongoing neuronal injury. Compared with early-NS, late-NS mainly damages the parenchyma with more severe clinical symptoms and worse prognosis. The late-NS had the highest level of CSF NFL and CSF sTREM2 in this study, suggesting that the levels of these two indicators may reflect the progress of NS and be related to the disability or severity of the disease. A positive correlation was found between CSF sTREM2 and CSF NFL, suggesting that microglial activation was associated with neuronal injury in *Treponema pallidum* infection in CNS. A previous study also found a positive correlation between CSF sTREM2 and CSF NFL in patients with HIV infection (8).

We found a moderate correlation between the levels of CSF sTREM2 and CSF NFL with age in syphilis/non-NS patients. The brain displays an increasing microglia activation and neuronal injury with normal aging. Aging can result in CSF sTREM2 and NFL release (29, 30). Gisslén et al. found that CSF sTREM2 and CSF NFL levels were strong correlated with age in HIV patients and normal controls (8). Suárez-Calvet M found that sTREM2 and CSF NFL levels were significant correlated with age in preclinical AD, AD dementia and the control group (11). The correlations between CSF sTREM2 and CSF NFL levels with age in syphilis/non-NS group were better than those in NS group, suggesting that microglial activation and neuronal injury in NS patients may obscure the effect of age on the CSF sTREM2 and CSF NFL levels (31).

This study has several limitations. In theory, the disease duration should be considered in this study, but it was difficult for the patients in this study especially those with late neurosyphilis, to give the exact time of syphilis infection. Our study was also limited by the relatively small sample size. A retrospective cross-sectional study was used in this study. A longitudinal study of CSF sTREM2 and CSF NFL in patients with NS could further evaluate their dynamic changes before and after penicillin treatment, and perhaps correlate their clinical significance of using sTREM2 or NFL as a prognostic or a therapeutic marker. Further studies are expected to address these issues.

## Conclusions

The study found that CSF concentrations of sTREM2 were higher in patients with NS compared to syphilis/non-NS patients, and peaked at late stage of NS, suggesting that CSF sTREM2 may be a useful marker of microglia activation in NS and play a role in the progression of NS. A positive correlation between the levels of CSF sTREM2 and CSF NFL indicates a certain linkage between microglial activation and neuronal injury in NS.

## Data Availability Statement

The datasets generated for this study are available on request to the corresponding author.

## Ethics Statement

The studies involving human participants were reviewed and approved by the Ethics committee of Beijing Ditan Hospital, Capital Medical University, Beijing, People's Republic of China (NO. 2019-026-001). The patients/participants provided their written informed consent to participate in this study.

## Author Contributions

WL, HC, WW, LY and XZ conceived and designed the study. WL, MJ, JG, and YX carried out the laboratory analyses. LY, WL, DX, and YH collected and organized the data and drafted the manuscript. WL, HC, and XZ performed the statistical analysis. All authors read, reviewed, and approved the final manuscript.

### Conflict of Interest

The authors declare that the research was conducted in the absence of any commercial or financial relationships that could be construed as a potential conflict of interest.
